# Role of the extracellular matrix in cell-cell communication: a new therapeutic target?

**DOI:** 10.1093/eurheartj/ehad061

**Published:** 2023-02-18

**Authors:** Thorsten Kessler, Hendrik B. Sager, Matthias Mann

**Affiliations:** 1 German Heart Centre Munich, Department of Cardiology, Technical University of Munich, Munich, Germany; 2 German Centre for Cardiovascular Research (DZHK e.V.), partner site Munich Heart Alliance, Munich, Germany; 3 Department of Proteomics and Signal Transduction, Max-Planck Institute of Biochemistry, Martinsried, Germany

Ischemic heart disease – the major cause of morbidity and death in the European Union and worldwide – is a consequence of coronary artery disease - the manifestation of atherosclerosis at the coronary vasculature - and myocardial infarction. In the past decades, lifestyle interventions, pharmacological treatment, and revascularization procedures markedly improved outcomes. It has been known for decades that atherosclerosis represents an inflammatory disease of the vascular wall. In the past, however, pharmacological treatment strategies mainly focused on platelet inhibition and statin treatment to lower LDL cholesterol and achieve further beneficial effects. Just recently, the CANTOS trial confirmed that anti-inflammatory treatments have beneficial effects in high-risk individuals^1^. All established strategies, however, have in common that they focus on circulating cells which are involved in thrombus formation (i.e., platelets) or recruited to atherosclerotic plaques (i.e., monocytes, neutrophils etc.). In constrast, plaque resident cells like endothelial cells (EC) or vascular smooth muscle cells (VSMC) that initiate and propagate local inflammatory processes are so far rather studied in experimental models. EC for instance, which represent the major barrier between tissues and the blood stream, were successfully targeted experimentally^2^ but the therapeutic potential of such approaches may be limited due to offsite effects that can be expected in other, non-diseased organs. These limitations can be overcome if we understand how local cells change their phenotype under disease conditions. In MATRICARD, a project funded by the European Research Council under the European Union’s Horizon 2020 research and innovation program within the Starting Grant scheme, we will focus on the role of the extracellular matrix (ECM) as a mediator of communication between different cell types in the vascular wall and the myocardium under inflammatory conditions (Fig. 1).

The ECM was mainly regarded as a microenvironment and scaffold for cells contributing to the bulk, shape and strength of tissues. In recent years, it has been recognized that the ECM is an active key player in the regulation of dynamic processes such as migration, proliferation and differentiation of resident cells^3^. There are important examples for an involvement of the ECM in cardiovascular diseases^4^: 1) Mutations in collagens, elastin and fibrillins were described to be causal in aortic aneurysm formation; 2) Genetic variation in genes encoding collagens, integrins, and matrix metalloproteinases was associated with atherosclerotic diseases; 3) Neointima formation after vascular injury is associated with massive alterations of the ECM including collagens, laminins, and matrix metalloproteinases^5^. In addition to structural alterations of the ECM, modulation of inflammation by ECM fragmentation has been described. Thrombospondin-1 is an example for an ECM protein with differential functions: whereas its degradation promotes neointima formation via delayed reendothelialization, a loss of thrombospondin-1 does not influence neointima formation secondary to vascular injury^6^.

The goal of MATRICARD is to get a closer look at ECM alterations which occur in acute and chronic cardiovascular inflammation and the downstream effects on other involved cell types. MATRICARD is an interdisciplinary project that will bring together clinician scientists and basic scientists. It is organized in four work packages (WP).

In WP1, we will explore the molecular changes that occur in acute and chronic inflammation. We will use transcriptome analyses to phenotype resident and recruited cells in different areas of interest. A holistic view and the observation of alterations in the ECM, however, furthermore requires a thorough analysis of the proteome. Fortunately, in recent years proteomics made great progress. It is not only possible to detect proteomic alterations in bulk tissue but also in different compartments. Quantitative detergent solubility profiling enables the analysis of the ECM proteome and has already been established in atherosclerosis^7^. The combination of high-end microscopy and mass spectrometry furthermore now also allows to investigate the proteome of single cells: Deep Visual Proteomics (DVP) is a technique that was first described in melanoma tissue and uses artificial intelligence-based image analysis to microdissect single cells from tissue sections for ultra-high-sensitivity mass spectrometry^8^. An application in cardiovascular diseases promises to provide insights on a new level. In WP2, we will investigate the role and the molecular mechanisms of targets identified in WP1. One target is the ECM protease ADAMTS-7 (Fig. 2).

Originally identified as a risk gene in coronary artery disease^9^, it was shown to modulate VSMC and EC phenotypes via degradation of different ECM proteins. The exact mechanisms in atherosclerosis remain incompletely understood but targeting ADAMTS-7 nevertheless represents an attractive therapeutic approach: a recent study found that a peptide-based vaccination against ADAMTS-7 was able to beneficially influence atherosclerotic plaque formation and vascular remodeling in mice^10^. Further ECM proteases but also their downstream targets could be promising candidates for mechanistic exploration. In WP3, we will therefore develop tools that enable us to identify modulators of our proteins of interest which can be used for therapeutic intervention.

MATRICARD is an experimental project and our findings will be limited by the artificial nature of experimental models in mice as a model organism. Therefore, it is important to compare findings from such experiments with available human data. The use of large-scale biobanks comprising genetic and phenotypic data of hundreds of thousands of individuals as well as biospecimen from individuals undergoing coronary bypass or carotid surgery for coronary or cerebrovascular atherosclerotic disease represents an excellent opportunity to validate findings and explore future translational approaches.

Taken together, the ECM represents a fascinating compartment with dynamic functions. We hope that within the next years, our group will be able to add novel insight into its function and identify novel putative targets to reduce the burden of cardiovascular inflammation and disease.

## Figures and Tables

**Figure 1:**
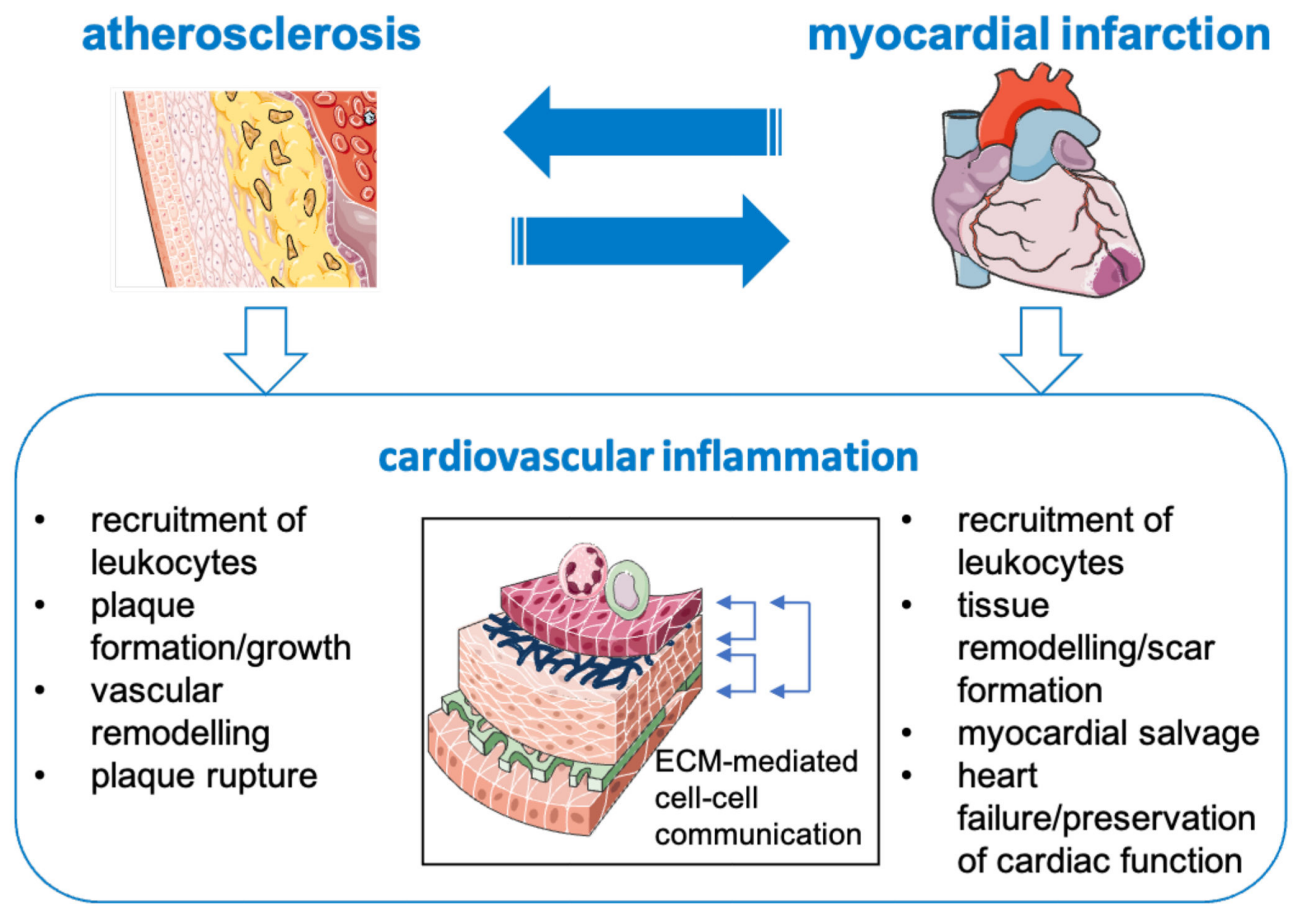
Acute and chronic cardiovascular inflammation. Coronary atherosclerosis is the prerequisite of myocardial infarction. On the other hand, myocardial infarction promotes progression of atherosclerosis. Atherosclerosis and myocardial infarction share pathophysiological features, e.g., recruitment of leukocytes. Other processes are unique for these acute and chronic entities of cardiovascular inflammation. MATRICARD aims at deciphering shared and unique pathways in the cell-cell communication via modulation of the extracellular matrix (ECM).

**Figure 2:**
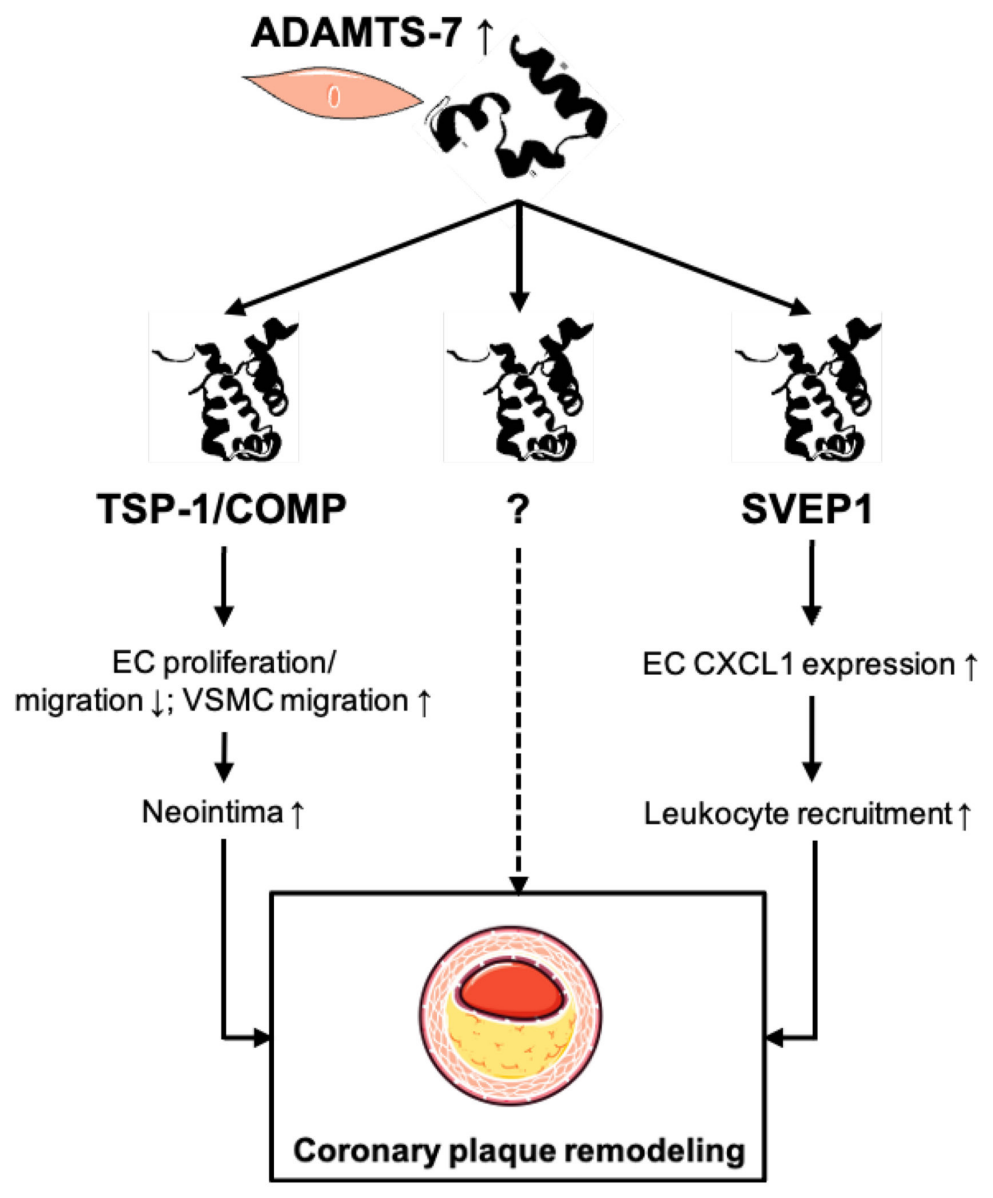
The extracellular matrix protease ADAMTS-7 as a role model for ECM-mediated cell-cell communication in cardiovascular disease. In the vasculature, ADAMTS-7 is mainly secreted by vascular smooth muscle cells (VSMC). In vascular remodeling (left), degradation of the ECM proteins thrombospondin-1 (TSP1) and cartilage oligomeric matrix protein (COMP) promotes neointima formation via reducing endothelial cell (EC) proliferation/migration and promoting VSMC migration. In atherosclerosis, its effect seems to be partly mediated via degradation of the ECM protein sushi, von Willebrand factor type A, EGF And pentraxin domain containing 1 (SVEP1; right). Loss of SVEP1 leads to enhanced cytokine release from EC, thereby promoting leukocyte recruitment from blood to plaque. Further thus far unknown mechanisms possibly contribute to the vascular effects of ADAMTS-7.

## References

[1] Ridker PM, Everett BM, Thuren T, MacFadyen JG, Chang WH, Ballantyne C, Fonseca F, Nicolau J, Koenig W, Anker SD, Kastelein JJP (2017). Antiinflammatory Therapy with Canakinumab for Atherosclerotic Disease. N Engl J Med.

[2] Sager HB, Dutta P, Dahlman JE, Hulsmans M, Courties G, Sun Y, Heidt T, Vinegoni C, Borodovsky A, Fitzgerald K, Wojtkiewicz GR (2016). RNAi targeting multiple cell adhesion molecules reduces immune cell recruitment and vascular inflammation after myocardial infarction. Sci Transl Med.

[3] del Monte-Nieto G, Fischer JW, Gorski DJ, Harvey RP, Kovacic JC (2020). Basic Biology of Extracellular Matrix in the Cardiovascular System, Part 1/4 JACC Focus Seminar. J Am Coll Cardiol.

[4] Barallobre-Barreiro J, Loeys B, Mayr M, Rienks M, Verstraeten A, Kovacic JC (2020). Extracellular Matrix in Vascular Disease, Part 2/4 JACC Focus Seminar. J Am Coll Cardiol.

[5] Wierer M, Werner J, Wobst J, Kastrati A, Cepele G, Aherrahrou R, Sager HB, Erdmann J, Dichgans M, Flockerzi V, Civelek M (2021). A proteomic atlas of the neointima identifies novel druggable targets for preventive therapy. Eur Heart J.

[6] Kessler T, Zhang L, Liu Z, Yin X, Huang Y, Wang Y, Fu Y, Mayr M, Ge Q, Xu Q, Zhu Y (2015). ADAMTS-7 inhibits re-endothelialization of injured arteries and promotes vascular remodeling through cleavage of thrombospondin-1. Circulation.

[7] Wierer M, Prestel M, Schiller HB, Yan G, Schaab C, Azghandi S, Werner J, Kessler T, Malik R, Murgia M, Aherrahrou Z (2017). Compartment-resolved Proteomic Analysis of Mouse Aorta during Atherosclerotic Plaque Formation Reveals Osteoclast-specific Protein Expression*. Mol Cell Proteomics.

[8] Mund A, Coscia F, Kriston A, Hollandi R, Kovács F, Brunner A-D, Migh E, Schweizer L, Santos A, Bzorek M, Naimy S (2022). Deep Visual Proteomics defines single-cell identity and heterogeneity. Nat Biotechnol.

[9] Schunkert H, König IR, Kathiresan S, Reilly MP, Assimes TL, Holm H, Preuss M, Stewart AFR, Barbalic M, Gieger C, Absher D (2011). Large-scale association analysis identifies 13 new susceptibility loci for coronary artery disease. Nat Genet.

[10] Ma Z, Mao C, Chen X, Yang S, Qiu Z, Yu B, Jia Y, Wu C, Wang Y, Wang Y, Gu R (2022). Peptide Vaccine Against ADAMTS-7 Ameliorates Atherosclerosis and Postinjury Neointima Hyperplasia. Circulation.

